# Comparison of Chemotherapeutic Activities of Rhodamine-Based GUMBOS and NanoGUMBOS

**DOI:** 10.3390/molecules25143272

**Published:** 2020-07-17

**Authors:** Nimisha Bhattarai, Mi Chen, Rocío L. Pérez, Sudhir Ravula, Robert M. Strongin, Karen McDonough, Isiah M. Warner

**Affiliations:** 1 Department of Chemistry, Louisiana State University, Baton Rouge, LA 70803, USA; nimishabhattarai@gmail.com (N.B.); michen109@gmail.com (M.C.); rperez@lsu.edu (R.L.P.); sudheer.ravula@gmail.com (S.R.);; 2 Department of Chemistry, Portland State University, Portland, OR 97207, USA; strongin@pdx.edu; 3 AgCenter Biotechnology Labs, Louisiana State University, Baton Rouge, LA 70803, USA; KMcdonough@agcenter.lsu.edu

**Keywords:** rhodamine dyes, nanoGUMBOS, chemotherapeutic activity

## Abstract

Rhodamine derivatives have been widely investigated for their mitochondrial targeting and chemotherapeutic properties that result from their lipophilic cationic structures. In previous research, we have found that conversion of Rhodamine 6G into nanoGUMBOS, i.e., nanomaterials derived from a group of uniform materials based on organic salts (GUMBOS), led to selective chemotherapeutic toxicity for cancer cells over normal cells. Herein, we investigate the chemotherapeutic activity of GUMBOS derived from four different rhodamine derivatives, two bearing an ester group, i.e., Rhodamine 123 (R123) and SNAFR-5, and two bearing a carboxylic acid group, i.e., rhodamine 110 (R110) and rhodamine B (RB). In this study, we evaluate (1) relative hydrophobicity via octanol–water partition coefficients, (2) cytotoxicity, and (3) cellular uptake in order to evaluate possible structure–activity relationships between these different compounds. Intriguingly, we found that while GUMBOS derived from R123 and SNAFR-5 formed nanoGUMBOS in aqueous medium, no distinct nanoparticles are observed for RB and R110 GUMBOS. Further investigation revealed that the relatively high water solubility of R110 and RB GUMBOS hinders nanoparticle formation. Subsequently, while R123 and SNAFR-5 displayed selective chemotherapeutic toxicity similar to that of previously investigated R6G nanoGUMBOS, the R110 and RB GUMBOS were lacking in this property. Additionally, the chemotherapeutic toxicities of R123 and SNAFR-5 nanoGUMBOS were also significantly greater than R110 and RB GUMBOS. Observed results were consistent with decreased cellular uptake of R110 and RB as compared to R123 and SNAFR-5 compounds. Moreover, these results are also consistent with previous observations that suggest that nanoparticle formation is critical to the observed selective chemotherapeutic properties as well as the chemotherapeutic efficacy of rhodamine nanoGUMBOS.

## 1. Introduction

Lipophilic rhodamine derivatives have been widely investigated for chemotherapeutic applications due to their hydrophobic structures and cationic properties [1,2,3]. Several studies have demonstrated that the mitochondrial membrane in cancer cells is relatively more negative compared to the mitochondrial membrane in normal cells, enabling partially selective accumulation of cationic compounds such as rhodamine in cancer cell mitochondria [4,5]. Other studies have shown that in addition to ionic properties, hydrophobicity also plays a major role in such mitochondrial accumulation [6,7]. In this regard, lipophilic cations, due to their lipophilic and cationic properties, have been found to have significantly greater accumulation in cancer cells in contrast to normal cells, ultimately resulting in partially selective toxicity [3,5,8]. 

Rhodamine derivatives, in particular, have been widely investigated for their therapeutic properties since their hydrophobicities have been found to be nearly optimal for partially selective behavior in contrast to other triarylmethane dyes such as ethyl violet [2,7,9,10]. In this regard, several studies have shown that Rhodamine 123 (R123) has promising in vitro and in vivo therapeutic efficacy [11,12,13,14]. Additionally, rhodamine 110 (R110) and rhodamine B (RB) have been investigated for chemotherapeutic and in vitro imaging applications [15,16,17]. Other studies have compared the in vitro imaging of zwitterion R110 and the cation R123 in order to understand the relevance of structure and charge to cellular uptake. Interestingly, while R110 has poor cellular uptake, R123 exhibits promising chemotherapeutic imaging properties [11,18]. Additional studies have demonstrated cellular internalization of the protonated acid form of R110 and RB. However, the acid-base properties of the carboxylic acid functional group limit their potential therapeutic and imaging properties.

Rhodamine dyes are also known to preferentially accumulate in the mitochondria and block ATP production, causing cellular apoptosis. However, the carboxylic acid functional group of RB and R110 causes a reduction in mitochondrial pH, leading to minimal mitochondrial accumulation and decreased therapeutic potential [7,16]. Thus, while cationic dyes such as R123 serve as strong imaging agents for mitochondria and mitochondria-targeting therapeutic agents, the zwitterion structures of RB and R110 reduce use for imaging and chemotherapeutic applications.

Nanocarrier systems such as liposomes, polymers, and micelles have been investigated as intracellular delivery systems for enhancing internalization of hydrophobic drugs [19,20,21,22]. This increased cellular uptake is typically due to the nanoscale size of such particles that allows for rapid permeation into cells [23,24]. Our research group has developed nanoGUMBOS, i.e., nanomaterials derived from a **g**roup of **u**niform **m**aterials **b**ased on **o**rganic **s**alts (GUMBOS), that display selective chemotherapeutic properties [25,26,27,28]. In previous studies from our group, synthesis of nanoGUMBOS using rhodamine 6G, a lipophilic cation with known anticancer properties, led to selective chemotherapeutic toxicity of cancer cells relative to normal cells using the resulting nanomaterials under examined conditions [26]. In contrast to existing nanocarrier systems that typically consist of liposomes and polymers, nanoGUMBOS provide distinct advantages, such as ease of synthesis, as well as tunable toxicity. Intriguingly, nanoGUMBOS often serve as the drug, eliminating a need for detailed characterization for drug loading and release profiles as well [29,30,31]. Furthermore, tunable properties of these nanomaterials may provide a strategy to overcome drug resistance problems that arise with many existing chemotherapeutics.

In order to understand the role of cation structure of rhodamine derivatives on selective chemotherapeutic toxicity, our studies reported here provide data for evaluation and comparison of therapeutic properties for GUMBOS derived from two ester derivatives, rhodamines (R123 and SNAFR-5) [32], and two zwitterion rhodamines (R110 and RB). Relative hydrophobicities of these GUMBOS were characterized using octanol–water partition coefficients. Subsequently, these compounds were employed in vitro in order to examine their cellular uptake and therapeutic potential for MDA-MB-231 cancer cells. Lastly, these materials were employed in Hs578Bst normal cells to examine their relatively selective chemotherapeutic behavior. In aggregate, these studies provide further insight into an approach for rapid synthesis of selective nanomaterials for direct use as cationic drugs to minimize systemic toxicity.

## 2. Results

### 2.1. Synthesis and Characterization

**Figure 1:**
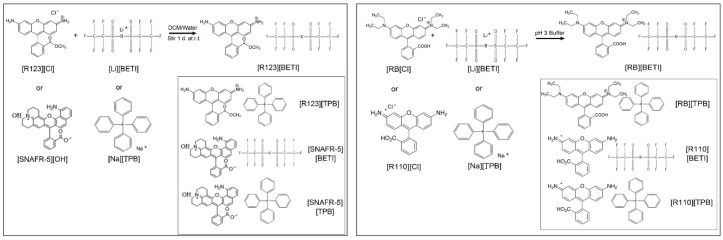
Synthesis of R123, SNAFR-5, RB, and R110 group of uniform materials based on organic salts (GUMBOS).

R123 and SNAFR-5 GUMBOS were synthesized using a simple metathesis reaction depicted in Figure 1. RB and R110 GUMBOS were synthesized using the single-phase aqueous reaction depicted in Figure 1. GUMBOS were characterized using electrospray mass spectrometry in order to confirm presence of the desired counter-ion (Table S1). Following synthesis of GUMBOS, hydrophobicity was examined using octanol–water partition coefficients (Kow) as reported in Table S2. The synthesized GUMBOS showed varying hydrophobicity based on anion variation. Examination of log K_ow_ for the cations R123 and SNAFR indicates that, for a constant cation and varying anions, the hydrophobicity trend beginning with the most hydrophobic compound was [X][BETI] ˃ [X][TPB] ˃ [X][Cl], where X represents the cation. The hydrophobicity trend for RB and R110 GUMBOS from most hydrophobic to most hydrophillic is [X][TPB] ˃ [X][BETI] ˃ [X][Cl]. Thus, these results demonstrate tunable hydrophobicity through counter-ion variation, which are quite similar to results obtained by Magut et al. [26].

**Figure 2:**
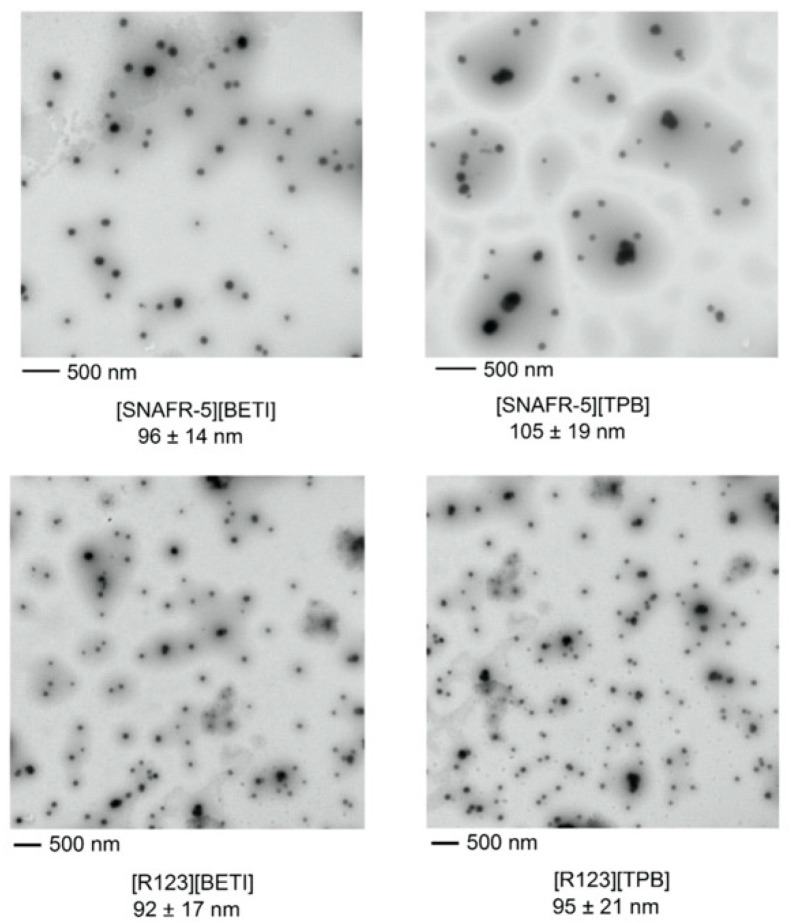
TEM images of R123 and SNAFR nanoGUMBOS.

Following examination of the hydrophobicity of these GUMBOS, nanoGUMBOS were synthesized using a simple reprecipitaiton method as described in the experimental section. TEM images in Figure 2 are a display of spherical nanoparticles with diameters of ~100 nm for both R123 and SNAFR. The zeta potential of the R123 and SNAFR nanoGUMBOS at physiological pH (7.4) is reported in Table 1. As shown in Table 1, zeta potentials for R123 and SNAFR nanoGUMBOS are around −17 mV, suggesting formation of relatively stable nanomaterials. These nanoparticles displayed a polydispersity below 0.2 when examined using dynamic light scattering (DLS), indicating formation of relatively monodispersed nanoparticles.

However, no distinct nanoparticles were observed for RB and R110 GUMBOS in contrast to R123 and SNAFR-5 GUMBOS. Thus, in order to further understand the lack of nanoparticle formation, the water solubilities of RB and R110 GUMBOS were examined at physiological pH. As shown in Table S3, the water solubility of these GUMBOS are significantly lower than that of the parent dyes. This is consistent with the increase in hydrophobicity observed for GUMBOS with respect to parent dyes. However, RB and R110 GUMBOS displayed a significantly higher water solubility than [R6G][BETI] and [R6G][TPB] GUMBOS that produced nanoGUMBOS in Magut et al. [26]. Rather, the water solubilities for these zwitterionic GUMBOS are relatively similar to the more hydrophilic GUMBOS found in Magut et al. [26]. In this regard, Magut et al. reported that nanoparticles were fabricated only from more hydrophobic GUMBOS, such as [R6G][BETI] and [R6G][TPB]. Thus, lack of nanoparticle formation can be attributed to the relatively high water solubility of the RB and R110 GUMBOS.

### 2.2. Spectroscopic Properties

**Figure 3:**
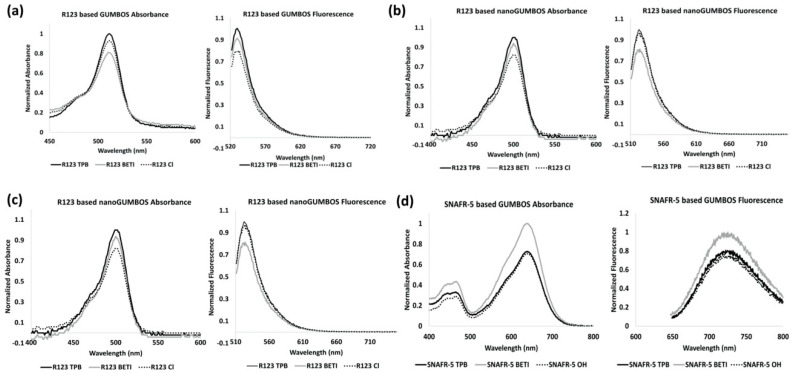
(**a**) Absorbance and fluorescence of R123-based GUMBOS in DMSO; (**b**) Absorbance and fluorescence of R123-based nanoGUMBOS in water; (**c**) Absorbance and fluorescence of SNAFR-based GUMBOS in DMSO; (**d**) Absorbance and fluorescence of SNAFR-based GUMBOS in DMSO.

In addition to synthesis and characterization of GUMBOS and nanoGUMBOS, spectroscopic studies were performed to examine the optical behavior of these novel materials. As indicated in Figure 3a, no shift in absorbance and fluorescence emission peak maxima were observed for R123 GUMBOS and the parent dye [R123][Cl] in DMSO. Formation of R123 nanoGUMBOS in water led to a slight 10 nm blue shift; however, no peak shift was observed between nanoGUMBOS and the parent dye in water (Figure 3b). Similar results were found for RB and R110 GUMBOS and these data are presented in Figure S1a–d. Examination of absorbance and fluorescence emission of SNAFR-5-based GUMBOS presented in Figure 3c indicates no peak shift following formation of GUMBOS. This observation is consistent with that of R123-based GUMBOS described earlier. Interestingly, formation of nanoGUMBOS led to a significant peak shift for [SNAFR][TPB] nanoGUMBOS as compared to the parent dye, while no peak shift was observed for [SNAFR][BETI] nanoGUMBOS (Figure 3d). In this regard, the absorbance of [SNAFR][BETI] nanoGUMBOS and the [SNAFR][OH] parent dye displayed blue shifting in water as compared to absorbance in DMSO. In contrast, the absorbance of [SNAFR][TPB] nanoGUMBOS was further red shifted in water in comparison to its absorbance in DMSO. Examination of the fluorescence emission presented in Figure 3d suggests significantly diminished fluorescence intensity for [SNAFR][TPB] in comparison to [SNAFR][BETI] and [SNAFR][OH]. This is possibly due to J-aggregation within [R6G][TPB] nanoGUMBOS as reflected in the red-shifted absorbance [33]. In addition, the longer wavelength of [SNAFR][TPB] nanoGUMBOS in contrast to typical rhodamines suggests that these nanoparticles are suitable for use in photodynamic therapy [34].

### 2.3. In Vitro Chemotherapeutic Efficacy

**Figure 4:**
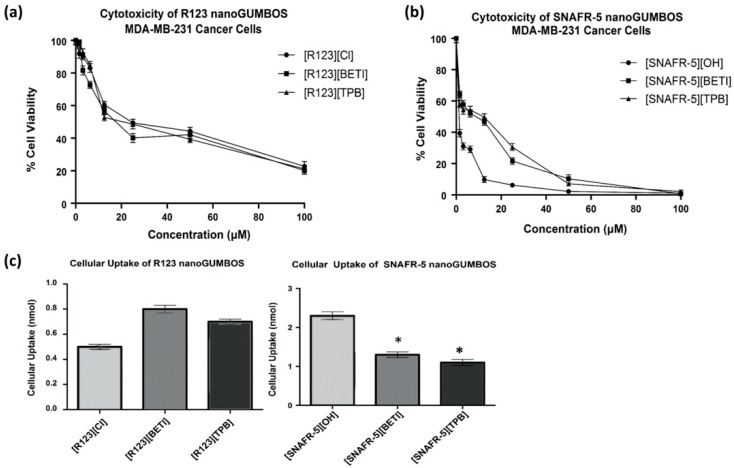
(**a**) Toxicity of R123 nanoGUMBOS towards MDA-MB-231 cancer cells; (**b**) Toxicity of SNAFR-5 nanoGUMBOS towards MDA-MB-231 cancer cells; (**c**) Cellular uptake of R123 nanoGUMBOS. (*) indicates significant difference in cellular uptake as compared to parent dye [SNAFR-5][OH] for respective nanoGUMBOS with *p* = 0.05.

These compounds were then employed in MDA-MB-231 breast cancer cells in order to examine their chemotherapeutic properties. Figure 4a,b are graphical representations of the cell viability of MDA-MB-231 breast cancer cells in the presence of R123 and SNAFR-5 based nanoGUMBOS treatment. The cell viability of MDA-MB-231 cancer cells with R123 nanoGUMBOS treatment was similar to the cell viability of these cells following treatment of the parent dye [R123][Cl] as shown in Figure 4a. This indicates that toxicity of the R123 nanoGUMBOS towards MDA-MB-231 cancer cells is of the same order of magnitude as the parent dye [R123][Cl]. IC_50_ concentrations presented in Table 2 represent the concentration at which 50% inhibition of cell proliferation was observed. [R123][TPB] and [R123][BETI] displayed IC_50_ concentrations of 17.4 µM and 20.6 µM, which are relatively similar to the IC_50_ concentration of [R123][Cl], 24.3 µM. While the IC_50_ values for R123 nanoGUMBOS are slightly lower than that of the parent dye, statistical analyses indicate no significant differences between the concentrations. [SNAFR5][BETI] and [SNAFR-5][TPB] displayed IC_50_ concentrations of 8.7 µM and 12.3 µM, respectively, while the parent dye [SNAFR-5][OH] displayed an IC_50_ concentration of 1.3 µM. These results suggest that formation of SNAFR-based nanoGUMBOS led to decreased toxicity in comparison with the parent dye.

Figure 4c presents data for cellular uptake of R123 and SNAFR nanoGUMBOS in MDA-MB-231 breast cancer cells after 5 h. incubation. The R123-based nanoGUMBOS displayed cellular uptake comparable to that of the parent dye [R123][Cl]. This is consistent with toxicity results that showed similar IC_50_ concentrations between R123 nanoGUMBOS and the parent dye. In contrast, SNAFR-5-based nanoGUMBOS displayed reduced cellular uptake as compared to the parent dye. This further corroborates decreased toxicity for SNAFR-5 nanoGUMBOS in comparison with the parent dye.

**Figure 5:**
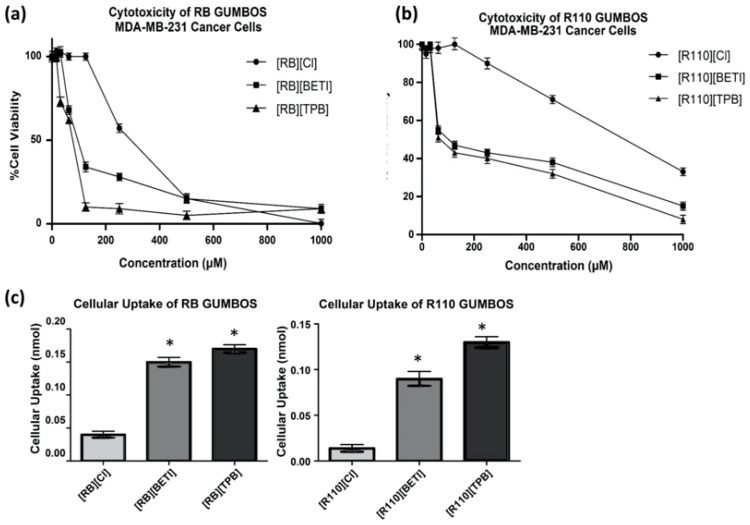
(**a**) Toxicity of RB GUMBOS towards MDA-MB-231 cancer cells; (**b**) Toxicity of R110 GUMBOS towards MDA-MB-231 cancer cells; (**c**) Cellular Uptake of R123 nanoGUMBOS. (*) indicates significant difference in cellular uptake as compared to parent dye [RB][Cl] or [R110][Cl] for respective nanoGUMBOS with *p* = 0.05.

Figure 5a,b are graphical representations of the toxicity studies of RB- and R110-based GUMBOS, respectively, towards MDA-MB-231 cancer cells. Intriguingly, a significant enhancement in toxicity for GUMBOS was observed as compared to the parent dye. As shown in graphs, the respective parent dyes remained relatively nontoxic until about 200 µM. However, GUMBOS displayed higher toxicity even at lower concentrations. In this regard, the IC_50_ values for the parent dyes [RB][Cl] and [R110][Cl] were 291 µM and 781 µM, respectively, while RB and R110 based GUMBOS displayed reduced IC_50_ values of 80–90 and 100–200 µM, respectively.

In order to further understand variations in IC_50_, cellular uptake of these compounds was then examined using MDA-MB-231 cells. As depicted in Figure 5c, RB and R110 GUMBOS displayed enhanced cellular uptake as compared to the respective parent dye. This improved cellular uptake is likely due to improved hydrophobic interactions of the dye with the phospholipid bilayer of the cell membrane. These results are consistent with conclusions drawn by Belostotsky, et al., where these authors indicate that variations in hydrophobicity can tune the interaction of a drug with the cell membrane [7]. However, all cellular uptakes of RB and R110 GUMBOS were significantly lower than R123 and SNAFR-5 nanoGUMBOS. This is consistent with a significantly higher IC_50_ concentrations for RB and R110 GUMBOS as compared to R123 and SNAFR-5 nanoGUMBOS.

As the IC_50_ of R123 and SNAFR nanoGUMBOS displayed a promising therapeutic efficacy towards MDA-MB-231 cancer cells, toxicities of these nanomaterials towards MCF7 breast cancer and MiaPaca pancreatic cancer cells were also evaluated. Table 2 is a comparison of the IC_50_ of R123 and SNAFR-5 based nanoGUMBOS towards MDA-MB-231, MCF7 and Mia-Paca cancer cell lines. While the R123 compounds displayed IC_50_ concentrations of 17–25 µM and 1–3 µM for MDA-MB-231 and MiaPaca cell lines, respectively, they displayed an IC_50_ above 100 µM for MCF7 cancer cells. These examinations suggest that the toxicity of nanoGUMBOS is greater towards the more aggressive MDA-MB-231 and Mia Paca cancer cells in contrast to the less aggressive MCF7 cancer cell line. Similar results were obtained for SNAFR-5-based nanoGUMBOS. However, in contrast to R123 compounds, the overall toxicity of SNAFR-5 was found to be greater. It is interesting to note that SNAFR-5-based nanoGUMBOS displayed less than 1 µM IC_50_ concentrations towards MiaPaca cancer cells, suggesting great therapeutic potential.

**Figure 6:**
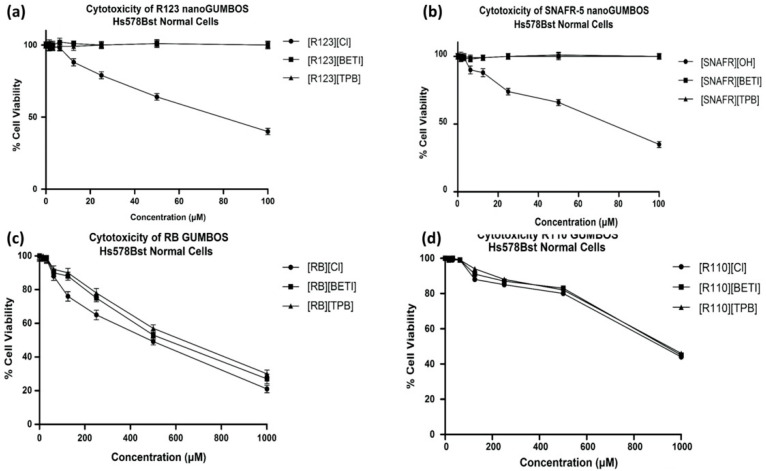
(**a**) Toxicity of R123 nanoGUMBOS towards Hs578Bst normal cells; (**b**) Toxicity of SNAFR-5 nanoGUMBOS towards Hs578Bst normal cells; (**c**) Toxicity of RB GUMBOS toward Hs578Bst normal breast cells; (**d**) Toxicity of R110 GUMBOS toward Hs578Bst normal breast cells.

Following application to the cancer cell lines cited above, all compounds were also evaluated using Hs578Bst normal breast cells to assess their selective chemotherapeutic properties. Figure 6a,b display toxicity of R123 and SNAFR-5 based nanoGUMBOS, respectively, towards Hs578Bst normal breast cells. Intriguingly, while both parent dyes [R123][Cl] and [SNAFR-5][OH] had slight toxicity towards normal cells, nanoGUMBOS from of these compounds led to selective toxicity toward cancer cells for these dyes under the conditions investigated. Furthermore, both parent dyes displayed a significantly higher IC_50_ for normal cells as compared to cancer cells. This partially selective behavior is consistent with findings reported by Belostotsky, et al., i.e., lipophilic rhodamine cations have enhanced cellular uptake in cancer cells as compared to normal cells due to electrostatic interactions with the negative mitochondrial membrane [7]. Furthermore, the selective behavior of nanoGUMBOS is most likely a result of energy-dependent pathways in contrast to diffusion. While both SNAFR and R123 parent compounds are relatively soluble in water and can use diffusion to internalize, nanoGUMBOS typically use active transport for internalization. Thus, this variation in internalization pathway, similar to that observed in our previous findings [27], is a plausible explanation for selective chemotherapeutic behavior of SNAFR and R123 nanoGUMBOS.

As shown in Figure 6c,d, R110 and RB GUMBOS displayed slight toxicity towards normal cells. Interestingly, these GUMBOS displayed significantly higher IC_50_ towards cancer cells as compared to normal cells, suggesting partial selectivity (Table 3). This is in contrast to the behavior of nanoGUMBOS derived from ester derivatives reported above that displayed complete selectivity. In this regard, while the ester derivative GUMBOS formed nanoGUMBOS in aqueous medium, the RB and R110 GUMBOS are water soluble and do not form nanoGUMBOS. As indicated earlier, the water solubility of RB and R110 GUMBOS was similar to that of the more hydrophilic GUMBOS previously reported by our group [26]. Intriguingly, these hydrophilic GUMBOS display toxicity towards normal cells, corroborating that the selective behavior observed for nanoGUMBOS derived from ester rhodamine derivatives is consistent with our previous conclusion that selectivity is due to nanoparticle formation. Thus, these results are consistent with results of our previous studies that demonstrate that selectivities of R6G nanoGUMBOS are dependent on nanoparticle formation [26].

## 3. Materials and Methods

### 3.1. Materials

Rhodamine B chloride, rhodamine 110 chloride, rhodamine 123, phosphate buffered saline (10× concentrate, 0.2 µM filtered), sodium tetraphenylborate [Na][TPB], dichloromethane (DCM), dimethylsulfoxide (DMSO), 1-octanol, sodium hydroxide (NaOH), citric acid monohydrate, and sodium phosphate dibasic were all purchased from Sigma-Aldrich (Milwaukee, WI, USA). Lithium bis(perfluoroethylsulfonyl)imide ([Li][BETI]) was obtained from Ionic Liquid Technologies (Tuscaloosa, Al, USA). Triply deionized water was obtained using an Aires High Purity Water System (Port Allen, LA, USA). The MTT (3-[4,5-Dimethylthiazol-2-yl]-2,5-diphenyltetrazolium bromide) cell viability assay was purchased from Promega Corporation (Madison, WI, USA). TEM grids were purchased from Ted Pella (Redding, CA, USA). SNAFR-5 dye was provided to us by one of our coauthors, Dr. Robert Strongin (Portland State University, Portland, OR, USA).

### 3.2. Synthesis of GUMBOS

Rhodamine 123 and SNAFR-5 GUMBOS were synthesized using a previously described biphasic ion-exchange reaction [26]. Briefly, a DCM solution of [R123][Cl] was mixed with aqueous [Li][BETI] or [Na][TPB] in a 1:1.2 molar ratio. This biphasic mixture was allowed to stir for 48 h at room temperature. Subsequently, the aqueous layer was removed, and the DCM layer was washed with deionized water to remove traces of [Li][Cl] or [Na][Cl]. The DCM layer was then evaporated and the product was dried in vacuo for 24 h to obtain the final product. RB and R110 were synthesized using a single-phase reaction scheme. Briefly, the rhodamine dye and the desired counter-ion, in the form of either [Li][BETI] or [Na][TPB], were both dissolved into a pH 3 citric acid phosphate buffer, and the solution was stirred for 15 min. The resultant pink precipitate was then centrifuged multiple times and washed with citric acid phosphate buffer each time to remove byproduct. The resultant product was then dried in vacuo. The final product was confirmed using ESI mass spectrometry in both positive and negative ion modes employing an Agilent ESI TOF 6230 mass spectrometer in the LSU mass spectrometry facility.

### 3.3. Synthesis of NanoGUMBOS

NanoGUMBOS were synthesized using a reprecipitation method [26]. Briefly, a DMSO solution containing GUMBOS was rapidly injected into cell media (2% volume ratio between DMSO and cell media) with pulsed ultrasonication for 5 min. NanoGUMBOS formed were then allowed to grow for 30 min, and the solution was diluted to 100 µM for TEM characterization and cell studies.

### 3.4. Octanol Buffer Partition Coefficients

Into a 20 mL vial, 1-Octanol is mixed with a pH 7.4 phosphate-citric acid buffer and stirred overnight. The two layers were separated and then a calibration curve was generated for each compound in 1-octanol using various concentrations. The phosphate-citric acid buffer was then added to one of the concentrations (C_i_) and this mixture was stirred for 48 h. Subsequently, absorbance in the octanol layer was measured and the concentration (C_o_) calculated using the calibration curves. Later, the equation C_i_ − C_0_ = C_w_ was used to calculate the aqueous concentration (C_w_). The octanol–water partition coefficient was then calculated using the equation K_ow_ = C_f_/C_w_.

### 3.5. Solubility Studies

Approximately fifty milliliters of water were added to three milligrams of GUMBOS. Absorbance measurements were then acquired over time until the absorbance reached a plateau. A calibration curve for an aqueous solution of GUMBOS was then generated at a soluble concentration, and the slope of the curve was used to calculate the solubility concentration. The solubility constant (Ksp) was then calculated using this concentration.

### 3.6. Spectroscopic Studies

Spectroscopic studies were preformed using a scanning spectrophotometer (UV-3101PC, Shimadzu, Columbia, MD, USA), and fluorescence emission was measured on HORIBA Spex Fluorolog-3-spectrofluorometer (model FL3-22TAU3, HORIBA, Piscataway, NJ, USA). Spectroscopic studies of all GUMBOS were performed using a 5 µM solution of GUMBOS in either DMSO or PBS Buffer. A reprecipitaiton method was used to synthesize the R123 and SNAFR-5 nanoGUMBOS for these studies. Briefly, a 1 mM solution of the GUMBOS in DMSO was reprecipitated using ultrasonication in phosphate buffered saline (2% DMSO/buffer ratio) for five minutes and aged for another 30 min to achieve a 5 µM nanoGUMBOS solution. All nanoGUMBOS solutions were sonicated for 1 min before analysis to ensure a homogenous mixture. RB and R110 GUMBOS were also prepared similarly for these studies.

### 3.7. Cell Culture

Hormone-independent breast adenocarcinoma (MDA-MB-231), hormone-dependent breast adenocarcinoma (MCF7), human pancreatic carcinoma (Mia-Paca), and normal human fibroblast cell lines were purchased from American Tissue Culture Collection (ATCC, Manassas, VA, USA). Cell lines were cultured to 90% confluence using ATTC guidelines for cell culture prior to experimentation.

### 3.8. Cell Viability Studies

A 96 well plate was seeded with 5000 cells/well and incubated for 24 h to allow attachment. Serial dilution from 100 µM to 0 µM was performed for each compound. These compounds were then incubated into the cells for 48 h, followed by MTT assay to determine cell viability. Firstly, 15 µL of the MTT dye solution was incubated in the cells for 3 h. The MTT dye reacts with NADPH present in live cells to form an insoluble purple formazan product. Subsequently, 100 µL of a stop solution was added to solubilize this product and halt the enzymatic reaction between NADPH and MTT. Cells were then incubated with stop solution for 1 h. The absorbance of the formazan was then measured at 570 nm using a microplate spectrophotometer. Cell viability is reported as the ratio between experimental groups and a control normalized to 100%. All measurements were performed in triplicate measurements to obtain standard error, and the reported cell viability is the average of these measurements. The IC_50_ was calculated using the formula:

$$\frac{\left(\left(0.5-\left(f\left(a\right)\right)\times \left(b-a\right)+\left(f\left(b\right)-f\left(a\right)\right)\times a\right)\right)}{\left(f\left(b\right)-f\left(a\right)\right)}$$

where a is the concentration where the cell viability is above 50%, b is the concentration where the cell viability is below 50%, and *f*(*a*) and *f*(*b*) are respectively the cell viability percentages *0.01 at concentration a and b respectively.

### 3.9. Cellular Uptake

For studies of cellular uptake, 200,000 cells were seeded in a 35 mm petri dish and then incubated at 37 °C overnight. These cells were then incubated with a 12.5 µM solution of nanoGUMBOS for 5 h. An untreated control containing no drug was used as a reference. Subsequently, the nanoGUMBOS solution was removed and cells were incubated with 3 mL of DMSO for 5 h until no cells were visually present when using a microscope. The absorbance of the DMSO solution was then measured using the untreated control as a reference. A calibration curve was generated employing a set of standards for each GUMBOS ranging from 1–10 µM. Cellular uptake was then calculated as nanomoles of compound internalized.

### 3.10. Microscopy

Briefly, 10,000 MDA-MB-231 cancer cells were seeded onto a 35 mm glass bottom petri dish and incubated overnight at 37 °C. Then, 20 nM of mitotracker was incubated with these cells for 30 minutes. Subsequently, a 25 nM nanoGUMBOS solution was incubated in the cells for 30 minutes. Finally, these cells were washed several times with buffer and imaged using a 40× dipping objective on a Leica Brightfield Microscope.

### 3.11. Statistical Analysis

A *t*-test was performed to determine significant differences between the IC_50_ concentrations and cellular uptakes of tested GUMBOS and nanoGUMBOS using *p* = 0.05 (95% confidence level).

## 4. Conclusions

Results reported here demonstrate the tunable hydrophobicity, solubility, and photophysical properties of GUMBOS through structural and counter-ion variations. Synthesis of GUMBOS from ester derivatives, rhodamines R123, and SNAFR-5, led to enhanced hydrophobicity in comparison to the respective parent dyes, ultimately leading to nanoparticle formation in aqueous medium. In contrast, the carboxylic acid rhodamines R110 and RB compounds led to formation of GUMBOS that were partially water soluble, resulting in lack of formation of nanoparticles. In vitro evaluation of these compounds suggest that these carboxylic acid derived rhodamine GUMBOS displayed nonselective behavior, while nanoGUMBOS from ester derivatives displayed selective chemotherapeutic properties, similar to previously reported studies [26,27,28]. Moreover, these findings further confirm that the concept of nanoGUMBOS can be used for various cationic dyes to generate an array of selective chemotherapeutics for combating the problem of systemic toxicity of current chemotherapeutics [35,36,37,38].

## Figures and Tables

**Table 1:** Zeta potential of R123 and SNAFR-5 nanoGUMBOS.

NanoGUMBOS	Zeta Potential
[R123][BETI]	−16.8 ± 1.1 mV
[R123][TPB]	−16.5 ± 1.4 mV
[SNAFR-5][BETI]	−17.4 ± 0.8 mV
[SNAFR-5][TPB]	−16.9 ± 1.3 mV

**Table 2:** IC_50_ values for R123- and SNAFR-5-based nanoGUMBOS towards MDA-MB-231, MiaPaca, and MCF7 cancer cell lines.

	MDA-MB-231 IC_50_ (µM)	MiaPaca IC_50_ (µM)	MCF7 IC_50_ (µM)
[R123][BETI]	17.4 ± 3.7	1.6 ± 0.7	˃100
[R123][TPB]	20.6 ± 3.5	2.5 ± 0.9	˃100
[R123][Cl]	24.3 ± 2.2	3.1 ± 1.1	˃100
[SNAFR-5][BETI]	8.7 ± 1.8	0.66 ± 0.03	32.5 ± 1.1
[SNAFR-5][TPB]	12.2 ± 2.9	0.72 ± 0.02	26.7 ± 2.2
[SNAFR-5]	1.3 ± 0.5	0.13 ± 0.02	3.7 ± 0.7

**Table 3:** IC_50_ concentrations of RB and R110 GUMBOS towards MDA-MB-231 cancer and Hs578Bst normal cells.

Compound	MDA-MB-231 IC_50_ (μM)	Hs578Bst IC_50_ (μM)
[RB][BETI]	89.5 ± 3.4	540.3 ± 6.2
[RB][TPB]	77.5 ± 5.7	533.7 ± 3.3
[RB][Cl]	291.0 ± 1.2	500.2 ± 5.2
[R110][BETI]	159.5 ± 1.1	843.8 ± 4.9
[R110][TPB]	105.5 ± 3.1	850.2 ± 3.7
[R110][Cl]	791.2 ± 2.7	836.1 ± 5.3

## Supplementary Materials

The following are available online. Table S1: Results from ESI mass spectrometric characterization of GUMBOS. Table S2: Relative hydrophobicity of R123 and SNAFR-5 based GUMBOS; Figure S1 (a) Absorbance and Fluorescence of RB GUMBOS in DMSO; (b.) Absorbance and Fluorescence of R110 GUMBOS in DMSO; (c) Absorbance and Fluorescence of RB GUMBOS in PBS Buffer; (d) Absorbance and Fluorescence of R110 GUMBOS in PBS Buffer.
