# Supplementary material for: Comparison of Chemotherapeutic Activities of Rhodamine-Based GUMBOS and NanoGUMBOS
Source: Molecules. 2020 Jul 17;25(14):3272. doi: 10.3390/molecules25143272 (PMC7397155; doi:10.3390/molecules25143272)
Supplement: Supplementary file 1 [file molecules-25-03272-s001.pdf]

molecules

MDPI

Article

Comparison of Chemotherapeutic Activities of Rhodamine-Based GUMBOS and nanoGUMBOS

Nimisha Bhattarai [1,†], Mi Chen [1,‡], Rocío L. Pérez [1], Sudhir Ravula [1,§], Robert M. Strongin [2], Karen McDonough [3] and Isiah M. Warner [1,*]

[1] Department of Chemistry, Louisiana State University, Baton Rouge, LA 70803, USA; nimishabhattarai@gmail.com (N.B.); michen109@gmail.com (M.C.); rperez@lsu.edu (R.L.P.); sudheer.ravula@gmail.com (S.R.);

[2] Department of Chemistry, Portland State University, Portland, OR 97207, USA; strongin@pdx.edu

[3] AgCenter Biotechnology Labs, Louisiana State University, Baton Rouge, LA 70803, USA; KMcdonough@agcenter.lsu.edu

* Correspondence: iwarner@lsu.edu; Tel.: 225-578-2829; Fax: 225-578-3971

† Present address - Department of Biochemistry and Molecular Biology, School of Medicine, Tulane University, New Orleans, LA 70118.

‡ Present address – Pharmaceutical Product Development, 3230 Deming Way, Middleton, WI, 53562, USA.

§ Present address – Department of Oral and Craniofacial Biology, Louisiana State University Health Sciences Center, New Orleans, LA 70112, USA.

Academic Editors: Po-Yu Chen, Jing-Fang Huang and Yen-Ho Chu
Received: 27 May 2020; Accepted: 14 July 2020; Published: date

Table S1. Results from ESI mass spectrometric characterization of GUMBOS.

Compound	Positive Mode		Negative Mode	
	Theoretical (*m/z*)	Actual (*m/z*)	Theoretical (*m/z*)	Actual (*m/z*)
[R123][BETI]	345.4	345.6	381.1	381.4
[R123][TPB]	345.4	345.5	319.3	319.2
[SNAFR-5][BETI]	458.3	458.2	381.1	381.2
[SNAFR-5][TPB]	458.3	458.2	319.3	319.1
[RB][TPB]	444.2	444.4	319.3	319.5
[RB][BETI]	444.2	44.43	381.1	381.3
[R110][TPB]	331.8	331.9	319.3	319.4
[R110][BETI]	331.8	331.7	381.1	381.4

Table S2. Relative hydrophobicity of R123 and SNAFR-5 based GUMBOS.

Compound	Log K_{ow}
[R123][BETI]	1.1
[R123][TPB]	0.8
[R123][Cl]	0.25
[SNAFR-5][BETI]	1.4
[SNAFR-5][TPB]	1.2
[SNAFR-5]	0.28
[RB][TPB]	1.6
[RB][BETI]	1.25
[RB][ClI]	1.1
[R110][TPB]	0.30
[R110][BETI]	0.32

[R110][Cl]	0.22

Table S3. Water solubility of RB and R110 GUMBOS.

Compound	Solubility (mol/L)	Dissociation Constant (mol²/L²)
[RB][TPB]	1.7×10^{-5}	1.7×10^{-10}
[RB][BETI]	1.3×10^{-5}	2.9×10^{-10}
[RB][ClI]	3.1×10^{-2}	9.8×10^{-4}
[R110][TPB]	4.1×10^{-5}	7.8×10^{-10}
[R110][BETI]	2.8×10^{-5}	1.7×10^{-10}
[R110][Cl]	$>7.0 \times 10^{-4}$	$>4.6 \times 10^{-7}$

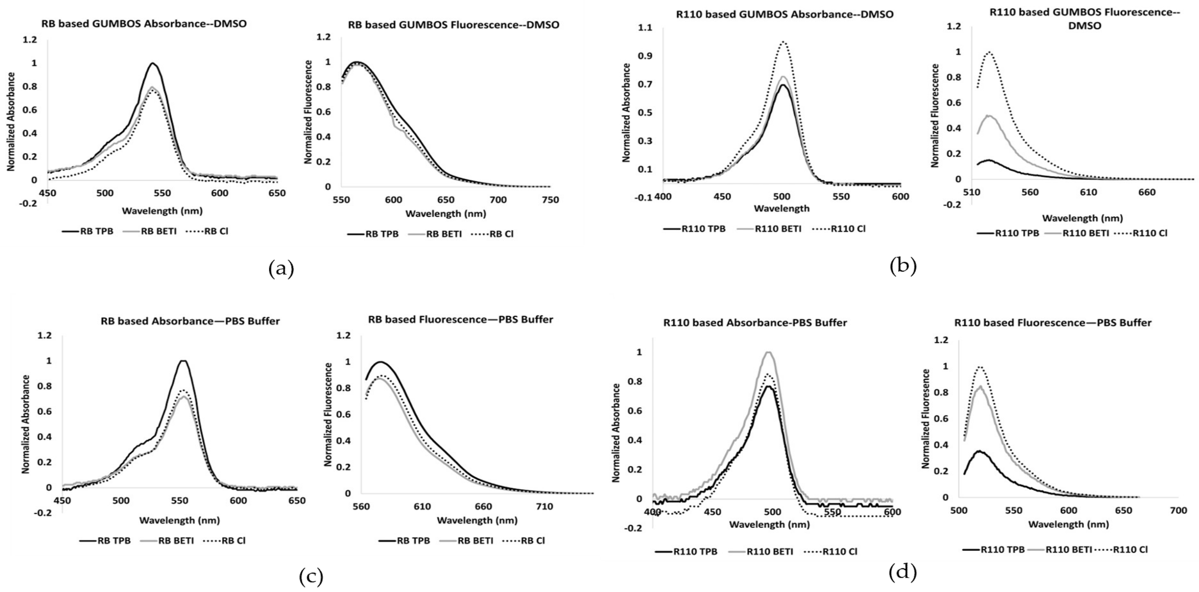

Figure S1. (**a**) Absorbance and Fluorescence of RB GUMBOS in DMSO; (**b**) Absorbance and Fluorescence of R110 GUMBOS in DMSO; (**c**) Absorbance and Fluorescence of RB GUMBOS in PBS Buffer; (**d**) Absorbance and Fluorescence of R110 GUMBOS in PBS Buffer.
